# Supplementary material for: Comparison between bupivacaine-lidocaine, dexamethasone mixture and bupivacaine alone for motor recovery after axillary brachial plexus block in distal radius surgery: A prospective randomized trial
Source: PLoS One. 2025 Apr 7;20(4):e0321087. doi: 10.1371/journal.pone.0321087 (PMC11975137; doi:10.1371/journal.pone.0321087)
Supplement: Attachment — Submitted filename: PONE-27 Dec.pdf [file pone.0321087.s003.pdf]

PLOS ONE

Comparative Motor Recovery in Axillary Brachial Plexus Block for Distal Radius Surgery: A Prospective, Randomized Controlled Trial of Bupivacaine-Lidocaine-Dexamethasone Mixture vs. Bupivacaine Alone

--Manuscript Draft--

**Manuscript Number:**	PONE-D-24-39685
**Article Type:**	Clinical Trial
**Full Title:**	Comparative Motor Recovery in Axillary Brachial Plexus Block for Distal Radius Surgery: A Prospective, Randomized Controlled Trial of Bupivacaine-Lidocaine-Dexamethasone Mixture vs. Bupivacaine Alone
**Short Title:**	Motor Recovery in Axillary Brachial Plexus Block for Distal Radius Surgery
**Corresponding Author:**	Busara Sirivanasandha<br>Mahidol University Faculty of Medicine Siriraj Hospital<br>Bangkok, THAILAND
**Keywords:**	Axillary brachial plexus block; Motor blockade; Perineural dexamethasone
**Abstract:**	Background<br><br>Brachial plexus block with long-acting local anesthetics can result in prolonged motor blockade, known as “dead arm,” which can cause discomfort and anxiety in patients. This study aimed to compare the recovery time of motor blockade between bupivacaine and a mixture of bupivacaine-lidocaine-dexamethasone in axillary brachial plexus block.<br><br>Methods<br><br>A prospective, randomized, double-blinded controlled trial was conducted with 70 patients scheduled for distal end radius fixation under axillary brachial plexus block. A local anesthetic mixture group (LA-mixture group) received a 21 ml mixture of 0.2% bupivacaine with 1.2% lidocaine and 5 mg of dexamethasone (n = 35). A bupivacaine group received 20 ml of 0.5% bupivacaine with 1 ml of normal saline (n = 35). Patient-reported outcomes were assessed for the duration of motor blockade, sensory blockade, pain after surgery, and satisfaction.<br><br>Results<br><br>The demographic data were similar between the two groups. The mean times for recovery of hand grips and sensation were 13.5 ± 7.3 and 12.6 ± 6.2 hours in the LA-mixture group and 15.3 ± 6.7 and 14.6 ± 6.2 hours in the bupivacaine group (P = 0.467 and 0.766, respectively). Pain scores were not significantly different between the two groups, but the incidence of rebound pain was lower in the LA-mixture group.<br><br>Conclusion<br><br>From the patients’ perspective, the bupivacaine-lidocaine-dexamethasone mixture provided similar block characteristics to bupivacaine alone in axillary brachial plexus block. However, the incidence of rebound pain appeared to be lower in the mixture group.
**Order of Authors:**	Rawee Jongkongkawutthi
	Paweenus Rungwattanakit
	Pathom Halilamien
	Suppachai Poolsuppasit
	Busara Sirivanasandha
**Additional Information:**	

Powered by Editorial Manager® and ProduXion Manager® from Aries Systems Corporation

Question	Response
**Financial Disclosure**<br><br>Enter a financial disclosure statement that describes the sources of funding for the work included in this submission. Review the submission guidelines for detailed requirements. View published research articles from *PLOS ONE* for specific examples.<br><br>This statement is required for submission and **will appear in the published article** if the submission is accepted. Please make sure it is accurate.<br><br>**Funded studies**<br>Enter a statement with the following details:<br>• Initials of the authors who received each award<br>• Grant numbers awarded to each author<br>• The full name of each funder<br>• URL of each funder website<br>• Did the sponsors or funders play any role in the study design, data collection and analysis, decision to publish, or preparation of the manuscript?<br><br>Did you receive funding for this work?	The author(s) received no specific funding for this work.
**Competing Interests**<br><br>Use the instructions below to enter a competing interest statement for this submission. On behalf of all authors, disclose any competing interests that could be perceived to bias this work—acknowledging all financial support and any other relevant financial or non-financial competing interests.<br><br>This statement is required for submission and **will appear in the published article** if the submission is accepted. Please make sure it is accurate and that any funding sources listed in your Funding Information later in the submission form are also	The authors have declared that no competing interests exist.

declared in your Financial Disclosure statement.<br><br>View published research articles from *PLOS ONE* for specific examples.<br><br>**NO authors have competing interests**<br><br>Enter: *The authors have declared that no competing interests exist.*<br><br>**Authors with competing interests**<br><br>Enter competing interest details beginning with this statement:<br><br>*I have read the journal's policy and the authors of this manuscript have the following competing interests: [insert competing interests here]*<br><br>* typeset	
**Ethics Statement**<br><br>Enter an ethics statement for this submission. This statement is required if the study involved:<br><br>• Human participants<br>• Human specimens or tissue<br>• Vertebrate animals or cephalopods<br>• Vertebrate embryos or tissues<br>• Field research<br><br>Write "N/A" if the submission does not require an ethics statement.<br><br>General guidance is provided below. Consult the submission guidelines for detailed instructions. **Make sure that all information entered here is included in the Methods section of the manuscript.**	The study protocol was approved by the Siriraj Institutional Review Board (approval number: Si 265/2020; approval date: 3 April 2020, under Assoc. Prof. Siriporn Pitimana-Aree, M.D., Chairperson of the Siriraj Institutional Review Board). All patients provided written informed consent.

Powered by Editorial Manager® and ProduXion Manager® from Aries Systems Corporation

**Format for specific study types**<br><br>**Human Subject Research (involving human participants and/or tissue)**<br>• Give the name of the institutional review board or ethics committee that approved the study<br>• Include the approval number and/or a statement indicating approval of this research<br>• Indicate the form of consent obtained (written/oral) or the reason that consent was not obtained (e.g. the data were analyzed anonymously)<br><br>**Animal Research (involving vertebrate animals, embryos or tissues)**<br>• Provide the name of the Institutional Animal Care and Use Committee (IACUC) or other relevant ethics board that reviewed the study protocol, and indicate whether they approved this research or granted a formal waiver of ethical approval<br>• Include an approval number if one was obtained<br>• If the study involved *non-human primates*, add *additional details* about animal welfare and steps taken to ameliorate suffering<br>• If anesthesia, euthanasia, or any kind of animal sacrifice is part of the study, include briefly which substances and/or methods were applied<br><br>**Field Research**<br><br>Include the following details if this study involves the collection of plant, animal, or other materials from a natural setting:<br>• Field permit number<br>• Name of the institution or relevant body that granted permission	
**Data Availability**<br><br>Authors are required to make all data underlying the findings described fully available, without restriction, and from the time of publication. PLOS allows rare exceptions to address legal and ethical concerns. See the PLOS Data Policy and FAQ for detailed information.	Yes - all data are fully available without restriction

A Data Availability Statement describing where the data can be found is required at submission. Your answers to this question constitute the Data Availability Statement and **will be published in the article**, if accepted.<br><br>**Important:** Stating 'data available on request from the author' is not sufficient. If your data are only available upon request, select 'No' for the first question and explain your exceptional situation in the text box.<br><br>Do the authors confirm that all data underlying the findings described in their manuscript are fully available without restriction?	
**Describe where the data may be found in full sentences. If you are copying our sample text, replace any instances of XXX with the appropriate details.**<br><br>• If the data are **held or will be held in a public repository**, include URLs, accession numbers or DOIs. If this information will only be available after acceptance, indicate this by ticking the box below. For example: *All XXX files are available from the XXX database (accession number(s) XXX, XXX.*).<br>• If the data are all contained **within the manuscript and/or Supporting Information files**, enter the following: *All relevant data are within the manuscript and its Supporting Information files.*<br>• If neither of these applies but you are able to provide **details of access elsewhere**, with or without limitations, please do so. For example:<br><br>*Data cannot be shared publicly because of [XXX]. Data are available from the XXX Institutional Data Access / Ethics Committee (contact via XXX) for researchers who meet the criteria for access to confidential data.*<br><br>*The data underlying the results presented in the study are available from (include the name of the third party*	All relevant data are within the manuscript.

Powered by Editorial Manager® and ProduXion Manager® from Aries Systems Corporation

and contact information or URL).<br>• This text is appropriate if the data are owned by a third party and authors do not have permission to share the data.<br><br>* typeset	
Additional data availability information:	

Manuscript

Click here to access/download;Manuscript;Main manuscript - Distal Radius 18092024.docx

Clinical trial

Comparative Motor Recovery in Axillary Brachial Plexus Block for Distal Radius Surgery: A Prospective, Randomized Controlled Trial of Bupivacaine- Lidocaine-Dexamethasone Mixture vs. Bupivacaine Alone

Rawee Jongkongkawutthi, Paweenus Rungwattanakit, Pathom Halilamien, Suppachai Poolsuppasit, Busara Sirivanasandha*

Department of Anesthesiology, Faculty of Medicine Siriraj Hospital, Mahidol University, Bangkok, Thailand

* Correspondence to:

Email address: busarasiri@gmail.com (B. Sirivanasandha)

Registered at the Thai Clinical Trials Registry (TCTR ID: TCTR20200114003) on 10 January 2020.

ABSTRACT

Background

Brachial plexus block with long-acting local anesthetics can result in prolonged motor blockade, known as "dead arm," which can cause discomfort and anxiety in patients. This study aimed to compare the recovery time of motor blockade between bupivacaine and a mixture of bupivacaine-lidocaine-dexamethasone in axillary brachial plexus block.

Methods

A prospective, randomized, double-blinded controlled trial was conducted with 70 patients scheduled for distal end radius fixation under axillary brachial plexus block. A local anesthetic mixture group (LA-mixture group) received a 21 ml mixture of 0.2% bupivacaine with 1.2% lidocaine and 5 mg of dexamethasone (n = 35). A bupivacaine group received 20 ml of 0.5% bupivacaine with 1 ml of normal saline (n = 35). Patient-reported outcomes were assessed for the duration of motor blockade, sensory blockade, pain after surgery, and satisfaction.

Results

The demographic data were similar between the two groups. The mean times for recovery of hand grips and sensation were 13.5 ± 7.3 and 12.6 ± 6.2 hours in the LA-mixture group and 15.3 ± 6.7 and 14.6 ± 6.2 hours in the bupivacaine group (P = 0.467 and 0.766, respectively). Pain scores were not significantly different between the two groups, but the incidence of rebound pain was lower in the LA-mixture group.

Conclusion

From the patients' perspective, the bupivacaine-lidocaine-dexamethasone mixture provided similar block characteristics to bupivacaine alone in axillary brachial plexus block. However, the incidence of rebound pain appeared to be lower in the mixture group.

Keywords: Axillary brachial plexus block; Motor blockade; Perineural dexamethasone

Background

Regional anesthesia is commonly performed for ambulatory hand surgery. Existing data show that regional anesthesia offers several advantages compared with general anesthesia during recovery after ambulatory hand surgery, including reduced opioid and antiemetic consumption and a shortened stay in the postanesthesia care unit [1-4].

The unpleasant feeling after a brachial plexus block is known as a "dead arm" or "prolonged motor block." Patients, especially those undergoing ambulatory procedures, experience difficulties performing routine activities involving their arms below the shoulder for an extended period when using long-acting local anesthetics. This consequence may impact the balance of elderly patients and increase the risk of falling, particularly in those with a history of fractured distal end of the radius due to falls [5-7]. Selecting appropriate local anesthetics for peripheral nerve blocks is crucial.

Bupivacaine is an effective local anesthetic for both central and peripheral nerve blocks, particularly in areas where ropivacaine is not accessible. Although bupivacaine is frequently used to provide postoperative analgesia due to its prolonged duration of action, it may not be suitable for ambulatory surgery. On the other hand, lidocaine has a shorter onset and duration of action, but patients may experience rebound pain when its effects wear off. Noncompliant bridging therapy is believed to be the leading cause of rebound pain after the peripheral nerve block subsides, especially in cases of extensive and dense blocks that increase the likelihood of a "dead arm" and rebound pain [7]. Dexamethasone is a commonly used adjuvant that provides prolonged analgesia while reducing rebound pain [8-10].

To obtain a rapid onset and sustained duration of anesthesia suited for surgery, we created a mixture of 1.2% lidocaine and 0.2% bupivacaine, with the addition of 5 mg of dexamethasone as an adjuvant. The total volume of the combination was 21 mL. This

combination aimed to decrease rebound pain and extend analgesia but not to impair motor function.

Our objective was to compare two local anesthetic mixtures: 1.2% lidocaine with 0.2% bupivacaine and dexamethasone 5 mg (total volume of 21 ml) versus 0.5% bupivacaine with normal saline (total volume of 21 ml). We evaluated the motor block duration as the primary outcome, as well as the sensation duration, time to moderate pain, numeric pain rating scale, and patient satisfaction score. We hypothesized that the local anesthetic mixture would result in a shorter motor block duration than bupivacaine alone for patients undergoing ultrasound-guided axillary brachial plexus block.

Materials and methods

Study design and population

This study was a single-center, prospective, double-blind, randomized controlled trial. It was approved by the Siriraj Institutional Review Board (approval number Si265/2020) and registered at the Thai Clinical Trials Registry (TCTR ID: TCTR20200114003)), registered 10 January 2020, https://www.thaiclinicaltrials.org/show/TCTR20200114003. The study was conducted on patients aged 20–85 years with American Society of Anesthesiologists physical status I–III who were scheduled to undergo elective open reduction and internal fixation of the distal radius at Siriraj Hospital between May 2020 and August 2021. The inclusion criteria for this study were patients with acute closed distal radius fractures who were undergoing an operation under axillary brachial plexus blockade. The following were the exclusion criteria:

- Body weight of less than 35 kg.
- History of chronic kidney disease with an estimated glomerular filtration rate of less than 50 ml/min/1.73 m2.
- Uncontrolled diabetes with a hemoglobin A1C level greater than 7.5%.
- Contraindication to regional anesthesia.
- Cognitive impairment.
- Preexisting neuropathy.
- Neurological deficit in the upper extremities.
- Known allergies to study medications.

Patients who declined to participate were also excluded from the study.

Randomization and blinding

A total of 70 patients were recruited and randomized into two groups: a local anesthetic mixture group (LA-mixture group) and a bupivacaine group. Randomization was performed in blocks

of four using a computerized random number generator in block of 4 (www.randomization.com), and the results were kept in opaque envelopes. Patients in the LA-mixture group (n = 35) received 21 ml of 1.2% lidocaine with 0.2% bupivacaine and dexamethasone 5 mg for the axillary brachial plexus block. Patients in the bupivacaine group (n = 35) were administered 20 ml of 0.5% bupivacaine with 1 ml of normal saline solution. The patients and the assessor were blinded to the treatment allocations.

Anesthetic protocol

This study was conducted in both inpatient and outpatient settings. Before the operative date, eligible patients were briefed about the research objectives and experimental methods via telephone, and they provided written consent on the day of their operation. Standard monitoring (pulse oximetry, noninvasive blood pressure monitoring, and electrocardiogram) was performed, and intravenous access was established in the nonsurgical upper extremity. Supplemental oxygen was administered via a nasal cannula at a rate of 2–3 L/min. Sedation was induced using intravenous midazolam (0.01–0.02 mg/kg) and/or fentanyl (0.5–1 mcg/kg).

For both study groups, an anesthesiologist with expertise in regional anesthesia performed an ultrasound-guided axillary brachial plexus block. Patients were positioned supine with the shoulder abducted and the elbow flexed in the pre-anesthetic block area. The axilla was sterilized, and an ultrasound probe was used to visualize the axillary artery in a short-axis view. A lidocaine skin wheal was raised, followed by the injection of the local anesthetic using an in-plane ultrasound technique. In the LA-mixture group, a total of 21 ml of 1.2% lidocaine with 0.2% bupivacaine and dexamethasone 5 mg was injected (12 ml of 2% lidocaine, 8 ml of 0.5% bupivacaine, and 1 ml of dexamethasone). In the bupivacaine group, 20 ml of 0.5% bupivacaine was injected with 1 ml of normal saline solution (total: 21 ml).

In the operating room, once the cast was removed from the fractured arm, the block's efficacy was validated by a decrease in cold and pin-prick sensation along the distribution of the radial, medial, ulnar, and musculocutaneous nerves compared with the contralateral arm. Motor blockade was evaluated by inability to perform both the hand grip and elbow flexion. Patients received an intravenous propofol infusion (50–100 mcg/kg/min) to provide mild to moderate sedation throughout the procedure. In the event of failed regional anesthesia, general anesthesia with a laryngeal mask airway was performed.

During the postoperative period, all patients followed the standard oral postoperative regimen. The regimen included paracetamol (500 mg) or paracetamol with codeine (500/8mg) (1 tablet, every 6 hours as needed); nonsteroidal anti-inflammatory drugs such as ibuprofen (400 mg, every 8 hours), celecoxib (200 mg, every 12 hours), or etoricoxib (90 mg, once daily); aescin (Reparil, 1 tablet three times per day); and tramadol or intravenous morphine for pain relief as needed.

Outcomes and measurements

The primary outcome was the duration of the motor block, as measured by the time after surgery until patients felt their motor power had recovered to preoperative levels. Patients were asked to maintain a block diary, noting when they first regained hand movement (time to motor recovery), how long the sensory block lasted, and when they first experienced moderate pain (numeric rating scale ≥ 4) at the surgical site. A single-blinded assessor, a research team member, contacted the patients on the evening of the surgery and again the following evening to collect data and check for complications. This standardized approach ensured consistent questioning and interpretation. The interview assessed how long it would take to restore motor power to pre-deployment block levels. If a patient's axillary brachial plexus block recovered during sleep, the block duration was considered to be when they awoke the following day.

The secondary outcomes were the time to the first instance of moderate pain or normal sensation, peak pain scores, average pain scores, pain medication usage, patient satisfaction scores, and the patient's preferred duration for motor recovery after the block. A pain score increase of more than two points indicated rebound pain [11].

Sample size

Prior research on the duration of motor recovery and the brachial plexus block found that the duration of a motor block from 0.5% bupivacaine was 14 ± 5.55 hours [12, 13]. Additionally, our pilot study revealed a mean difference of 5 hours (standard deviation of 6 hours) in motor recovery between 0.5% bupivacaine and an LA mixture. Our power analysis indicated that, with an anticipated 10% dropout rate, 32 patients per group (64 in total) would be required to demonstrate a statistically significant 5-hour difference in motor power recovery time. The power analysis was based on a 90% power to detect a 5-hour difference with an alpha of 0.05. In total, the study aimed to enroll 70 patients.

Statistical analysis

All statistical analyses were conducted using IBM SPSS Statistics, version 20 (IBM Corp, Armonk, NY, USA). Data normality was assessed using histograms. Continuous data are presented as the means with standard deviations (SDs) or medians with interquartile ranges [IQRs]. Categorical data are reported as counts and percentages. Comparisons between the two groups utilized the chi-square test for grouped data, the unpaired t-test for normally distributed continuous data, and the Mann–Whitney U test for non-normally distributed continuous data.

Results

Between May 2020 and August 2021, 98 patients with closed distal radius fractures who met the criteria were enrolled. Eighteen patients (18.4%) were excluded for various reasons: eight had concomitant injuries; four displayed glomerular filtration rates below 50 mL/min/1.73 m²; two had hemoglobin A1C levels exceeding 7.5%; two were allergic to an analgesic as per the protocol; one presented acute coronary symptoms presurgery; and one showed signs of median nerve compression. An additional ten patients (10.2%) were excluded due to the unavailability of research staff, resulting in a final sample of 70. No patients dropped out after randomization (Fig 1.).

Table 1 Demographic data

	**LA-mixture group (n=35)**	**Bupivacaine group (n=35)**	***P***
Age (years)	54.6 (±16.2)	58.1 (±15.2)	0.348
Female gender (n)	21 (60%)	27 (77.1%)	0.122
BMI (kg/m^2)	23.62 (±4.53)	23.65 (±4.15)	0.979
ASA classification (n)			0.560
I	17 (48.6%)	15 (42.9%)	
II	15 (42.9%)	14 (40.0%)	
III	3 (8.6%)	6 (17.1%)	
Underlying diseases (n)			
DM	2 (5.7%)	6 (17.1%)	0.259
HT	10 (28.6%)	10 (28.6%)	1.000
CAD	0	1 (2.9%)	1.000
CVA	0	3 (8.6%)	0.239
Other	17 (48.6%)	18 (51.4%)	0.811
GFR (mL/min/1.73 m²)	88.61 (±23.77)	90.09 (±16.66)	0.766
Time to surgery (days)	11 [9–16]	9 [6–13]	0.069
Preoperative pain score (NRS 0–10)	0 [0–2]	1 [1–2.5]	0.275
Ambulatory surgery (n)	17 (48.6%)	15 (42.9%)	0.631
Operation on dominant hand (n)	14 (40.0%)	18 (51.4%)	0.332

ASA, American Society of Anesthesiologists; BMI, body mass index; CAD, coronary artery disease; CVA, cerebrovascular accident; DM, diabetes mellitus; GFR, glomerular filtration rate; HT, hypertension; NRS, numeric rating scale

Data are shown as the means (± SD), n (%), and median [interquartile range; IQR]

There were no statistically significant differences between the LA-mixture group and the bupivacaine group in terms of patient demographics, the interval between injury and surgery, or preoperative pain scores (Table 1). Of the surgeries, 32 (45.7%) were conducted as ambulatory procedures, and 32 (45.7%) involved the dominant hand. The distribution of these factors was similar between the two groups.

Table 2. Intraoperative data

	**LA-mixture group (n=35)**	**Bupivacaine group (n=35)**	***P***
Propofol (mg/kg/min)	61.04 (±28.21)	57.02 (±27.99)	0.552
Fentanyl (mcg/kg)	0.86 (±0.26)	1.08 (±0.40)	0.034*
Midazolam (mg)	0 [0–0]	0 [0–1]	0.077
Tourniquet time (min)	87.77 (±27.04)	85.94 (±24.12)	0.766
Operative time (min)	94.66 (±23.96)	90.34 (±25.18)	0.465

Data are shown as the means (± SD) and median [IQR]

Operation duration and tourniquet periods were comparable between the groups. The choice of intraoperative sedative drugs was consistent between groups, except for fentanyl dosage. The LA-mixture group had a mean intraoperative fentanyl dosage of 0.86 ± 0.26 mcg/kg compared to the bupivacaine group's 1.08 ± 0.4 mcg/kg (P = 0.034), as indicated in Table 2.

One patient in the bupivacaine group had to be converted to a general anesthetic with a laryngeal mask airway during the bone fixation stage due to pain and movement. For this patient, the total administered amounts of propofol, midazolam, and fentanyl were 110 mg, 1.5 mg, and 100 mcg, respectively. However, after the operation, a postoperative reassessment revealed a complete sensory and motor block, negating the necessity for study dropout. The duration of postoperative motor and sensory block recovery for this patient, who had general anesthesia, was also documented.

Table 3. Postoperative outcomes

	LA-mixture group (n=35)	Bupivacaine group (n=35)	*P*
Motor recovery (hours)			
Hand	14.14 (±6.92)	15.31 (±6.47)	0.467
Elbow	13.19 (±6.87)	13.54 (±6.68)	0.833
Time to first pain (hours)	12.69 (±6.18)	14.61 (±6.15)	0.766
Pain score (NRS 0–10)			
Average pain	3 [2–5]	4 [2–5]	0.261
Worst pain	5 [3–7]	5 [3–8]	0.092
Rebound pain (n)	3 (8.6%)	11 (31.4%)	0.017*
Satisfaction score (NRS 0–10)			
Motor block	8.00 (±1.70)	7.83 (±1.34)	0.641
Sensory block	8.29 (±1.79)	8.17 (±1.38)	0.766
Overall	8.46 (±1.63)	8.23 (±1.31)	0.520
Desired recovery speed (hours)	0 [0–0]	0 [0–1]	0.418
Block wear-off during sleep (n)	12 (34.3%)	18 (51.4%)	0.147

NRS, numeric rating scale

Data are shown as the means (± SD) and median [IQR], n (%)

In the postoperative period (Table 3), no significant differences were observed between the two groups in terms of motor and sensory recovery. For the LA-mixture group, recovery times for hand grip and elbow flexion were 14.14 ± 6.92 hours and 13.19 ± 6.87 hours, respectively. In contrast, the bupivacaine group exhibited recovery times of 15.31 ± 6.47 hours and 13.54 ± 6.68 hours (P = 0.467 and 0.833, respectively). The 95% confidence interval was -4.37 to 2.02.

Neither group showed significant differences in either average or worst pain scores. In the LA-mixture group, the median average pain score was 3 [IQR 2–5], and the worst pain score was 5 [IQR 3–7]. In the bupivacaine group, the corresponding scores were 4 [IQR 2–5] and 5 [IQR 3–8].

The LA-mixture group had a significantly lower incidence of rebound pain: only 3 patients (8.6%), compared to 11 patients (31.4%) in the bupivacaine group (P = 0.017). All

patients expressed a desire to regain hand movement as soon as possible after surgery. Patient satisfaction scores were 8.46 ± 1.63 in the LA-mixture group and 8.23 ± 1.31 in the bupivacaine group (P = 0.520). For the patient who needed general anesthesia, motor recovery times for the hand and elbow were 760 and 160 minutes, respectively. The time to first pain for this patient was 1000 minutes.

Table 4. Postoperative medications

	**LA-mixture group (n=35)**	**Bupivacaine group (n=35)**	***P***
Paracetamol	28 (80.0%)	31 (88.6%)	0.324
NSAIDs	34 (97.1%)	33 (94.3%)	1.000
Aescin (Reparil)	20 (57.1%)	27 (77.1%)	0.075
Paracetamol with codeine	5 (14.3%)	11 (31.4%)	0.088
Tramadol	8 (22.9%)	3 (8.6%)	0.101
Morphine	6 (17.1%)	10 (28.6%)	0.255

NSAIDs, nonsteroidal anti-inflammatory drugs

Data are shown as n (%)

Regarding postoperative medication, there was no difference between the groups in terms of the number of patients who received paracetamol, nonsteroidal anti-inflammatory drugs, aescin (Reparil), paracetamol with codeine, tramadol, and intravenous morphine for severe pain after surgery (Table 4).

Discussion

In this randomized trial, we investigated the duration of the motor block after an axillary brachial plexus block under ultrasound guidance. We compared two groups: 1.2% lidocaine and 0.2% bupivacaine with dexamethasone 5 mg totaling 21 ml (the LA-mixture group) versus 0.5% bupivacaine with normal saline solution totaling 21 ml (the bupivacaine group). Our study found no statistically significant differences between the two groups in motor block duration, sensory block duration, or postoperative pain scores. The LA-mixture group showed a mean motor block recovery time of 14.1 hours for hand grip and 13.2 hours for elbow flexion. The bupivacaine group recorded longer mean recovery times of 15.3 hours for hand grip and 13.5 hours for elbow flexion ($P = 0.467$ and 0.833, respectively).

Several factors may account for these nonsignificant results. First, perineural dexamethasone's effect on motor blockade needs to be considered. Although the primary local anesthetic in the LA-mixture group was lidocaine, which typically wears off in 2–3 hours, adding dexamethasone may extend this period to 5 hours [14, 15]. Furthermore, 0.2% bupivacaine predominantly affects sensory function or pain control [16]. However, in our study, adding 5 mg perineural dexamethasone improved blocking efficiency, extending the motor block duration to 6–8 hours. A study by Aliste et al. reported that the addition of perineural dexamethasone to a mixture of 1% lidocaine and 0.25% bupivacaine increased block duration/analgesia by 4.0–4.7 hours, resulting in a total motor block time of 17.5 hours [10]. This extended motor block duration may be attributed to the synergistic effects of dexamethasone combined with lidocaine and bupivacaine, especially considering the higher concentration of bupivacaine used in their study compared to ours.

Dexamethasone is administered both intravenously and perineurally for pain management. Numerous studies have ascertained that dexamethasone can mitigate pain and prolong sensory block [17-20]. However, the impact of a prolonged motor block has recently

gained more attention. Consistent with the findings from several previous studies [10, 14, 21], our investigation found that dexamethasone increased the duration of the motor block when combined with 1.2% lidocaine. Nevertheless, this effect had not been adequately considered by previous researchers. We recommend that caution should be exercised when adding perineural dexamethasone to peripheral nerve blocks in the lower limb, especially for procedures that require early ambulation, such as femoral nerve block or popliteal nerve block. This is because the motor block effects may persist longer than anticipated and increase the risk of falls.

The tool used for evaluation was another factor that could have influenced the primary outcome. In this study, patient-reported outcomes were deemed appropriate for an ambulatory setting. Moreover, since the focus was on capturing patients' real-life feelings and opinions regarding the block, the precise duration of the block was not a critical factor, unlike in other studies. However, it is important to note that the accuracy of patient reports in pain and movement diaries can vary; even a feeling of numbness may be misinterpreted as weakness. To mitigate this potential source of error, we ensured that only one team member explained the assessment process before surgery and followed up with consistent postsurgery questions. Future trials should consider incorporating objective measurement tools, such as a hand grip dynamometer to measure pre- and postoperative muscle strength, or evaluating functional capabilities through activities such as grabbing a ball or demonstrating hand movements.

A third factor contributing to the inconsistencies observed was the wearing off of the blocking effect during sleep. In the LA-mixture group, 12 cases (34%) experienced block wear-off during sleep, compared to 18 cases (51%) in the bupivacaine group. This discrepancy could markedly distort the apparent motor block duration. It is worth noting that no patient woke up in the middle of the night in severe discomfort, as all patients were advised to take painkillers before bedtime.

Most participants in our study desired to regain motor function as quickly as possible after surgery. Fredrickson et al. [16] highlighted that the discomfort associated with an insensate arm often required a temporary halt in the continuous infusion of a high-concentration local anesthetic, even though the purpose of the anesthetic was to alleviate pain. While there were no significant differences in motor block duration between groups in our study, refraining from using dexamethasone and reducing the bupivacaine concentration to decrease motor block time might benefit ambulatory surgery patients by enabling them to return to routine activities more swiftly. It is worth noting that most participants reported high satisfaction levels. This is likely due to participants being informed beforehand about the possibility of prolonged motor block and instructed on arm care, reducing potential surprise or frustration about this side effect.

Other anesthesia techniques for distal end radius fixation include general anesthesia, intravenous regional anesthesia, the Bier block, and the wide-awake local anesthesia no-tourniquet (WALANT) block. The WALANT technique has recently gained traction as a safe and effective anesthetic option for various hand surgeries. Originally developed for soft tissue surgeries, the WALANT technique has since been extended to include bone procedures. This method allows for earlier intraoperative assessment and functional wrist recovery while eliminating the adverse effects of sedation [22-24]. However, the WALANT approach is contraindicated for patients who cannot undergo the awake procedure.

As anticipated, no significant difference in sensory duration was observed in our study. However, the incidence of rebound pain was significantly lower in the LA-mixture group than in the bupivacaine group (3.6% vs 31.4%, $P = 0.0017$). Given that rebound pain following upper limb blocks with tense and long-acting local anesthetics can be considerable [7], using a lower but appropriate concentration for the procedure and adding dexamethasone have been shown to minimize rebound pain across a spectrum of peripheral nerve blocks [17, 19, 20].

Due to lidocaine's faster onset than bupivacaine's, combining these local anesthetics may result in a quicker onset of a tense block than pure bupivacaine. If that is the case, it might explain why the mean intraoperative fentanyl consumption in the LA-mixture group was less than that in the bupivacaine group (0.86 ± 0.26 vs 1.08 ± 0.4 mcg/kg; P = 0.034). Conversely, the mean propofol and midazolam doses for sedation were not significantly different. Notably, one patient in the bupivacaine group necessitated general anesthesia, yet the block proved effective upon reassessment at the end of the procedure. The combination of short- and long-acting local anesthetics may be particularly beneficial in situations that require a rapid onset or adherence to a tight schedule. However, the onset of the local anesthetics combination may be determined by the concentration of lidocaine in the mixture [25, 26].

It is well established that the risk of local anesthetic systemic toxicity is higher with bupivacaine due to its high cardiac toxicity and narrow therapeutic window, which can lead to severe consequences [27]. To curtail these potential adverse side effects, the bupivacaine concentration was scaled down, and the medication was mixed with lidocaine, which has a broader therapeutic window. We demonstrated that the mix of 1.2% lidocaine, 0.2% bupivacaine, and 5 mg of dexamethasone achieved a similar duration of sensorimotor blockade as 0.5% bupivacaine alone while theoretically reducing the risk of local anesthetic toxicity. This mixture may present a safer alternative for blocking techniques requiring high doses or volumes of local anesthesia. However, this study observed no instances of local anesthetic systemic toxicity.

The study had several limitations. First, the research was conducted at a single center, potentially affecting the generalizability of the findings. Second, the ambulatory setting introduced challenges in data collection, especially in the realm of phone-based evaluations. This setting also posed challenges in ensuring patient compliance with postoperative medication and pain management protocols. Nevertheless, these challenges are unlikely to have

influenced the primary study outcomes, given that previous research has indicated similar efficacy among oral nonsteroidal anti-inflammatory drugs in perioperative pain reduction [28]. Third, the absence of a group that received a local anesthetic without dexamethasone makes it difficult to attribute observed outcomes exclusively to our chosen interventions. Although this design choice aimed to achieve comparable pain management outcomes, it may have inadvertently led to an underestimation of the influence of dexamethasone on motor blockade. Finally, this study's definition of rebound pain is not universally accepted [11, 17, 29]. The criterion we adopted for analysis in ambulatory cases was an increase in pain score of more than two points (11).

Conclusions

In summary, the combination of 1.2% lidocaine, 0.2% bupivacaine, and 5 mg dexamethasone is a viable alternative to 0.5% bupivacaine in ambulatory settings. This mix lessens rebound pain and possibly reduces the required dose of intraoperative fentanyl due to the combination's rapid onset of action. Nevertheless, the duration of the sensorimotor block for the ultrasound-guided axillary brachial plexus block remains the same.

Ethics approval statement: The study protocol was approved by the Siriraj Institutional Review Board (approval number: Si 265/2020; approval date: 3 April 2020, under Assoc. Prof. Siriporn Pitimana-Aree, M.D., Chairperson of the Siriraj Institutional Review Board) and registered with the Thai Clinical Trials Registry (TCTR ID: TCTR20200114003) on 10 January 2020, https://www.thaiclinicaltrials.org/show/TCTR20200114003. All patients provided written informed consent.

Funding: None

Data availability statement: All relevant data are within the manuscript.

Competing interests: The authors have declared that no competing interests exist.

Acknowledgements

The authors are indebted to Miss Julaporn Pooliam for assistance with the methodology, Mrs. Nichapat Thongkaew for her invaluable support with administrative work, and to Mr. David Park for thoroughly proofreading the manuscript.

Credit authorship contribution statement

Conceptualization: Rawee Jongkongkawutthi, Busara Sirivanasandha.

Data curation: Rawee Jongkongkawutthi, Paweenus Rungwattanakit, Suppachai Poolsuppasit, Pathom Halilamien.

Formal analysis: Rawee Jongkongkawutthi, Busara Sirivanasandha.

Methodology: Busara Sirivanasandha.

Investigation: Rawee Jongkongkawutthi, Paweenus Rungwattanakit, Suppachai Poolsuppasit, Pathom Halilamien.

Project administration: Busara Sirivanasandha.

Supervision: Busara Sirivanasandha.

Writing – original draft: Rawee Jongkongkawutthi, Busara Sirivanasandha.

Writing – review & editing: Busara Sirivanasandha.

References

1. McCartney CJ, Brull R, Chan VW, Katz J, Abbas S, Graham B, et al. Early but no long-term benefit of regional compared with general anesthesia for ambulatory hand surgery. Anesthesiology. 2004;101:461-7.
2. Rundgren J, Mellstrand Navarro C, Ponzer S, Regberg A, Serenius S, Enocson A. Regional or General Anesthesia in the Surgical Treatment of Distal Radial Fractures: A Randomized Clinical Trial. The Journal of bone and joint surgery American volume. 2019;101:1168-76.
3. Egol KA, Soojian MG, Walsh M, Katz J, Rosenberg AD, Paksima N. Regional anesthesia improves outcome after distal radius fracture fixation over general anesthesia. Journal of orthopaedic trauma. 2012;26:545-9.
4. Patel AA, Buller LT, Fleming ME, Chen DL, Owens PW, Askari M. National trends in ambulatory surgery for upper extremity fractures: a 10-year analysis of the US National Survey of Ambulatory Surgery. Hand (New York, NY). 2015;10:254-9.
5. Ganta A, Ding D, Fisher N, Lavery J, Jain S, Tejwani NC. Continuous Infraclavicular Brachial Block Versus Single- Shot Nerve Block for Distal Radius Surgery: A Prospective Randomized Control Trial. Journal of orthopaedic trauma. 2018;32:22-6.
6. Fredrickson MJ, Wolstencroft PJ, Chinchanwala S, Boland MR. Does motor block related to long- acting brachial plexus block cause patient dissatisfaction after minor wrist and hand surgery? A randomized observer- blinded trial. British journal of anaesthesia. 2012;109:809-15.
7. Sellbrant I, Karlsson J, Jakobsson JG, Nellgård B. Supraclavicular block with Mepivacaine vs Ropivacaine, their impact on postoperative pain: a prospective randomised study. BMC anesthesiology. 2021;21:273.

8. Chong AK, Tan DM, Ooi BS, Mahadevan M, Lim AY, Lim BH. Comparison of forearm and conventional Bier's blocks for manipulation and reduction of distal radius fractures. The Journal of hand surgery, European volume. 2007;32:57-9.

9. Barry GS, Bailey JG, Sardinha J, Brousseau P, Uppal V. Factors associated with rebound pain after peripheral nerve block for ambulatory surgery. British journal of anaesthesia. 2021;126:862-71.

10. Aliste J, Leurcharusmee P, Engsusophon P, Gordon A, Michelagnoli G, Sriparkdee C, et al. A randomized comparison between intravenous and perineural dexamethasone for ultrasound-guided axillary block. Canadian journal of anaesthesia = Journal canadien d'anesthesie. 2017;64:29-36.

11. Williams BA, Bottegal MT, Kentor ML, Irrgang JJ, Williams JP. Rebound pain scores as a function of femoral nerve block duration after anterior cruciate ligament reconstruction: retrospective analysis of a prospective, randomized clinical trial. Regional anesthesia and pain medicine. 2007;32:186-92.

12. Venkatesh RR, Kumar P, Trissur RR, George SK. A Randomized Controlled Study of 0.5% Bupivacaine, 0.5% Ropivacaine and 0.75% Ropivacaine for Supraclavicular Brachial Plexus Block. Journal of clinical and diagnostic research : JCDR. 2016;10:Uc09-uc12.

13. Ilham C, Bombaci E, Yurtlu S, Çolakoğlu S. Efficiency of levobupivacaine and bupivacaine for supraclavicular block: a randomized double-blind comparative study. Brazilian journal of anesthesiology (Elsevier). 2014;64:177-82.

14. Movafegh A, Razazian M, Hajimaohamadi F, Meysamie A. Dexamethasone Added to Lidocaine Prolongs Axillary Brachial Plexus Blockade. Anesthesia & Analgesia. 2006;102.

15. Biradar PA, Kaimar P, Gopalakrishna K. Effect of dexamethasone added to lidocaine in supraclavicular brachial plexus block: A prospective, randomised, double- blind study. Indian journal of anaesthesia. 2013;57:180-4.

16. Fredrickson MJ, Price DJ. Analgesic effectiveness of ropivacaine 0.2% vs 0.4% via an ultrasound- guided C5- 6 root/ superior trunk perineural ambulatory catheter. British journal of anaesthesia. 2009;103:434-9.

17. Muñoz-Leyva F, Cubillos J, Chin KJ. Managing rebound pain after regional anesthesia. Korean journal of anesthesiology. 2020;73:372-83.

18. Holmberg A, Hassellund SS, Draegni T, Nordby A, Ottesen FS, Gulestøl A, et al. Analgesic effect of intravenous dexamethasone after volar plate surgery for distal radius fracture with brachial plexus block anaesthesia: a prospective, double-blind randomised clinical trial(). Anaesthesia. 2020;75:1448-60.

19. Lee MJ, Koo DJ, Choi YS, Lee KC, Kim HY. Dexamethasone or Dexmedetomidine as Local Anesthetic Adjuvants for Ultrasound- guided Axillary Brachial Plexus Blocks with Nerve Stimulation. The Korean journal of pain. 2016;29:29-33.

20. Fang J, Shi Y, Du F, Xue Z, Cang J, Miao C, et al. The effect of perineural dexamethasone on rebound pain after ropivacaine single- injection nerve block: a randomized controlled trial. BMC anesthesiology. 2021;21:47.

21. Chong MA, Berbenetz NM, Lin C, Singh S. Perineural Versus Intravenous Dexamethasone as an Adjuvant for Peripheral Nerve Blocks: A Systematic Review and Meta-Analysis. Regional anesthesia and pain medicine. 2017;42:319-26.

22. Huang YC, Chen CY, Lin KC, Yang SW, Tarng YW, Chang WN. Comparison of Wide- Awake Local Anesthesia No Tourniquet With General Anesthesia With Tourniquet for Volar Plating of Distal Radius Fracture. Orthopedics. 2019;42:e93-e8.

23. Boukebous B, Maillot C, Castel LC, Donadio J, Boyer P, Rousseau MA. Wide awake local anesthesia no tourniquet (WALANT) versus axillary brachial plexus block for carpal tunnel release in a French public university hospital: Care pathways and operating room costs. Orthopaedics & Traumatology: Surgery & Research. 2023;109:103358.

24. Abitbol A, Merlini L, Masmejean EH, Gregory T. Applying the WALANT technique to surgical treatment of distal radius fractures. Hand surgery & rehabilitation. 2021;40:277-82.

25. Powell MF, Jarzombek KW, Venhuizen KJ, Tubinis MD, Morgan CJ, Frölich MA. Comparing Bupivacaine, Lidocaine, and a Combination of Bupivacaine and Lidocaine for Labor Epidural Activation: A Prospective, Randomized, Double-Blind Study. Asian journal of anesthesiology. 2019;57:55-60.

26. Maben D, Suresh A, Desai AK, Shetty S, Juturu U, Anand J. Efficacy of lidocaine vs combination of lidocaine and bupivacaine in management of maxillofacial trauma: a clinical comparative study. Clinical Oral Investigations. 2023;27:6613-7.

27. Bourne E, Wright C, Royse C. A review of local anesthetic cardiotoxicity and treatment with lipid emulsion. Local and regional anesthesia. 2010;3:11-9.

28. Rømsing J, Møiniche S. A systematic review of COX-2 inhibitors compared with traditional NSAIDs, or different COX-2 inhibitors for post-operative pain. Acta anaesthesiologica Scandinavica. 2004;48:525-46.

29. Hamilton DL. Rebound pain: distinct pain phenomenon or nonentity? British journal of anaesthesia. 2021;126:761-3.

Figure legend

Fig 1 CONSORT 2010 Flow Diagram

Fig 1 CONSORT 2010 Flow Diagram

Click here to access/download;Figure;Figure 1 CONSORT 2010 Flow Diagram 280824.jpg

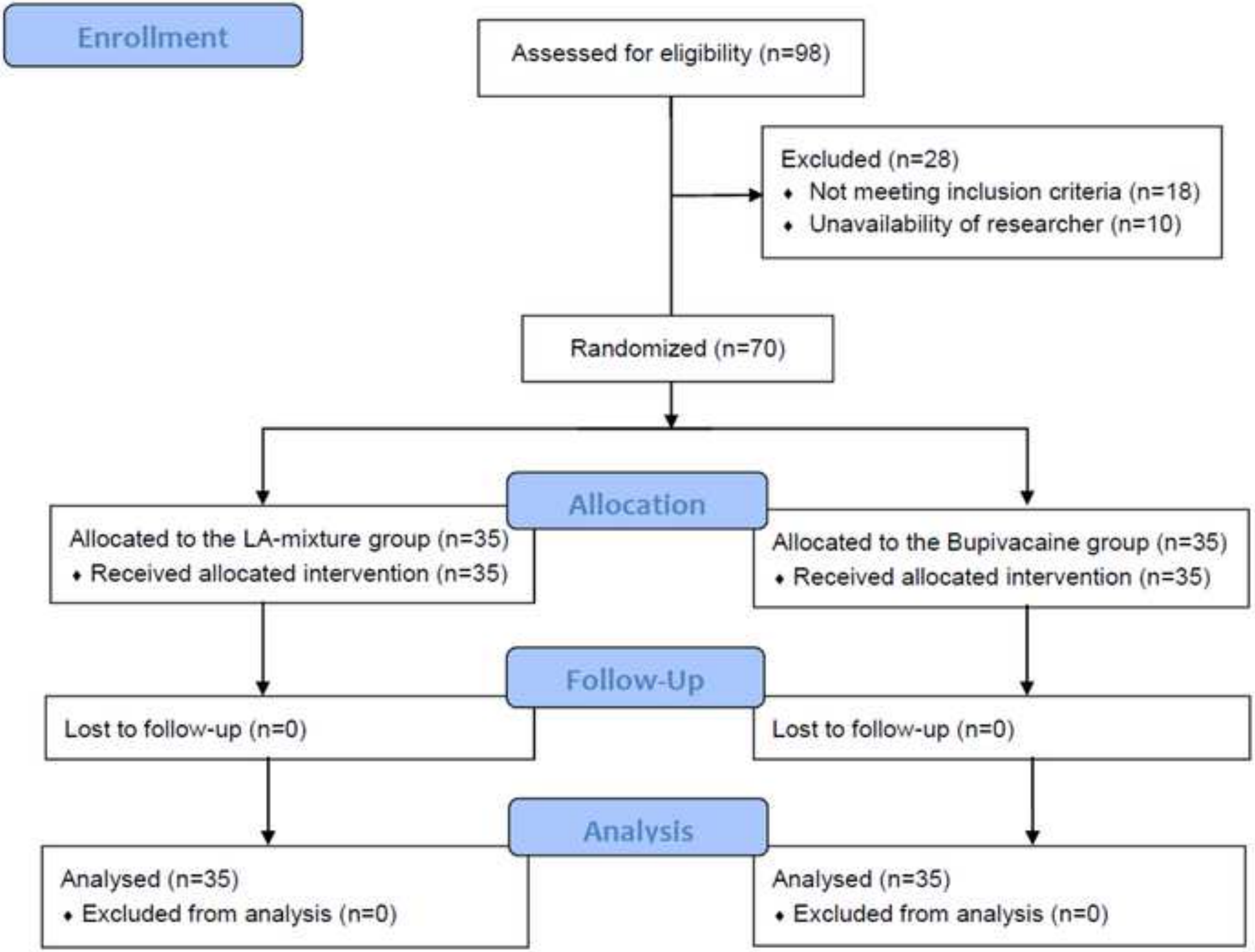

Click here to access/download
Supporting Information
Research proposal - IRB COA no 265_2020.pdf

Click here to access/download
Other
CONSORT 2010 - 8092024.pdf

Click here to access/download
Other
IRB_COA_265.2020.pdf
