# Supplementary material for: Comparison between bupivacaine-lidocaine, dexamethasone mixture and bupivacaine alone for motor recovery after axillary brachial plexus block in distal radius surgery: A prospective randomized trial
Source: PLoS One. 2025 Apr 7;20(4):e0321087. doi: 10.1371/journal.pone.0321087 (PMC11975137; doi:10.1371/journal.pone.0321087)
Supplement: Attachment — Submitted filename: Response to reviewer 07022025.docx [file pone.0321087.s004.docx]

**Manuscript number:** PONE-D-24-39685

**Manuscript title:** Comparison Between Bupivacaine-Lidocaine, Dexamethasone Mixture and Bupivacaine Alone for Motor Recovery After Axillary Brachial Plexus Block in Distal Radius Surgery: A Prospective Randomized Trial

**Journal name:** PLOS ONE

**Corresponding author:** Busara Sirivanasandha

**Reviewers' comments:**

Reviewer's Responses to Questions

**Comments to the Author**

1. Is the manuscript technically sound, and do the data support the conclusions?

The manuscript must describe a technically sound piece of scientific research with data that supports the conclusions. Experiments must have been conducted rigorously, with appropriate controls, replication, and sample sizes. The conclusions must be drawn appropriately based on the data presented.

Reviewer #1: Partly

Reviewer #2: Yes

Reviewer #3: Partly

2. Has the statistical analysis been performed appropriately and rigorously?

Reviewer #1: Yes

Reviewer #2: Yes

Reviewer #3: Yes

3. Have the authors made all data underlying the findings in their manuscript fully available?

The [PLOS Data policy](http://www.plosone.org/static/policies.action#sharing) requires authors to make all data underlying the findings described in their manuscript fully available without restriction, with rare exception (please refer to the Data Availability Statement in the manuscript PDF file). The data should be provided as part of the manuscript or its supporting information, or deposited to a public repository. For example, in addition to summary statistics, the data points behind means, medians and variance measures should be available. If there are restrictions on publicly sharing data—e.g. participant privacy or use of data from a third party—those must be specified.

Reviewer #1: Yes

Reviewer #2: Yes

Reviewer #3: Yes

4. Is the manuscript presented in an intelligible fashion and written in standard English?

PLOS ONE does not copyedit accepted manuscripts, so the language in submitted articles must be clear, correct, and unambiguous. Any typographical or grammatical errors should be corrected at revision, so please note any specific errors here.

Reviewer #1: Yes

Reviewer #2: Yes

Reviewer #3: Yes

5. Review Comments to the Author

Please use the space provided to explain your answers to the questions above. You may also include additional comments for the author, including concerns about dual publication, research ethics, or publication ethics. (Please upload your review as an attachment if it exceeds 20,000 characters)

**Reviewer #1:** This manuscript describes a prospective randomised controlled study on the comparison between the effect of bupivacaine alone or a combination of bupivacaine, lignocaine and dexamethasone on recovery from motor blockade following axillary block for hand surgery. The manuscript is well presented and the original premise is reasonable. However, there are several aspects to this study that render the manuscript not novel or adding significant value to current evidence base.

**First,** the concept of dense motor block is well known among clinicians who practise these blocks. Using 0.5% bupivacaine is uncommon for this reason and in many centres, the concentration of bupivacaine used is 0.25% and in some instances even 0.125%. It is well established that using low concentrations of bupivacaine reduce the incidence of a dense block. This doesn’t require the multiple drug combinations used in the LA mixture arm of this study, thus obviating the need for this study.

**Response:** Thank you for your valuable comment. At our institution, 0.5% bupivacaine is often used because it provides a faster onset of effect than lower dosages. Lower concentrations of bupivacaine provide an effective, albeit less dense and shorter-lasting, motor block compared to higher concentrations, but they have a considerable effect on onset time. The details have been added to the manuscript. (Page4, Line 59-68)

**Second,** the addition of dexamethasone is not sufficiently motivated in this manuscript. If dexamethasone is used to increase the duration of analgesia in the absence of extended motor block, then there should be another arm of the study that investigated the combination of lignocaine and 0.2% bupivacaine. Clinical experience suggests that motor blockade would have been shorter in duration in this third group. Further, the authors have presented evidence from previous research that dexamethasone can increase duration of motor block. This confuses the primary outcome measure for the sake of assessing reduction in rebound pain (a secondary outcome measure).

**Response:** Thank you for your valuable feedback. We admit that we did not expect dexamethasone to have such a significant influence on the primary outcome, as prolong motor blockage. We considered that a local anesthetic mixture without dexamethasone might not achieve an analgesic duration comparable to that of bupivacaine alone. As our objective was to provide rapid motor recovery while maintaining equivalent analgesic duration, we prioritized the inclusion of dexamethasone in the local anesthetic mixture to achieve this goal. We've already mentioned this issue in the discussion. (Page 16, line 275-286)

**Third,** rebound pain as described in the study is not defined and measured according to previously established methodology. Hence the findings from this study can not be reproduced in future studies. There is no indication in the manuscript of stratification of patients who may be at higher risk of rebound pain and therefore it is difficult to understand the results presented here.
**Response:** Thank you for your valuable feedback. Our definition of rebound pain was a pain score increase of higher than two points, as per Williams et al. We acknowledge that there is currently not a firm consensus on the formal definition and assessment of rebound pain. However, we wanted to demonstrate another disadvantage of bupivacaine alone: the potential of increased rebound pain to the local anesthetic mixture. (Page 5, line 73-77) Also the definition of diagnosis rebound pain in Outcome and Measurement. (Page9, line 162-163)

**Fourth**, there is some discrepancy between the statistical metrics presented in the table and in the text (rebound pain 3.6% and 8.6%, p values: 0.017 and 0.0017). These indicate either typographical errors or significant errors,

**Response:** Thank you for your helpful feedback. We acknowledge the typographical error. After rechecking all the statistics, we discovered that the data on rebound pain was incorrect. We've already corrected to " only 3 patients (8.6%), compared to 10 patients (28.6%) in the bupivacaine group (P = 0.031). (Page 13, line 241-242) (Page 14, Table 3) (Page 18, line 336)

**Finally,** the statistically significant difference between the dose of fentanyl required in the 2 groups is clinically insignificant. There are also several grammatical and typographical errors that need to be corrected.

**Response:** Thank you for your important comment. We’ve already corrected in the discussion part. (Page19, line 343-344)

**Reviewer #2:** The sample size and statistical analysis plan are simple and well stated. Presumably the significance level is set at 0.05. Please explain the asterisk in the footnote area of Tables 2 and 3. The outcomes are in Table 3 with the only statistically significant result being a comparison of proportion of rebound pain. All the tests in Table 3 are univariate. This prompts the question of multiple comparisons with a need for p-value adjustment. The authors should acknowledge this or at least tell why no adjustment is made.
The study limitations are outlined appropriately.

**Response:** Thank you for your valuable comment. We have clarified the meaning of the asterisk and the significance level in the footnotes of Tables 2 and 3 as “*p <0.05 indicates statistical significance.”

Table 3 shows a variety of outcomes, including rebound pain, which is defined as a difference of more than two points between worst and average pain. Furthermore, because rebound pain was a different dimension from the other outcomes, the p-value cannot be adjusted. (Page 14, Table 3)

**Reviewer #3:** i appreciate the authors efforts in conducting this trial that investigated the ability of local anesthetic mixture to avoid delayed motor recovery. i have the following comments:

1- although the title has all components of CONSORT checklist, but it is haphazardly written and needs to be arranged in a logical way to avoid confusion
suggestion:
comparison between Bupivacaine- Lidocaine, Dexamethasone Mixture vs. Bupivacaine Alone for motor recovery after Axillary Brachial Plexus Block in Distal Radius surgery; a prospective randomized trial.
**Response:** Thank you for your valuable comment. We revised and rewrote the title as “Comparison between Bupivacaine- Lidocaine, Dexamethasone Mixture vs. Bupivacaine Alone for motor recovery after Axillary Brachial Plexus Block in Distal Radius surgery; a prospective randomized trial.” (Page 1, line 3-5)

2- in abstract:
- it is better to replace (satisfaction) with the outcome that reported in the results and conclusion (incidence of pain rebound)
**Response:** Thank you for your valuable comment. We replaced patient satisfaction with the incidence of rebound pain. (Page 2, line 38-39)

- define the 1ry and 2ry outcomes in methods
**Response:** Thank you for your valuable comment. We revised and rewrote the Methods section of the abstract to clearly define the primary and secondary outcomes as “The primary outcome was the duration of motor blockade. Secondary outcomes included the duration of sensory blockade, postoperative pain score and the incidence of rebound pain.” (Page 2, line 31-33)

- add only the significant p value to the results.
**Response:** Thank you for your valuable comment. We have revised the Results section in the Abstract to include only the significant p-values as “(8.6% and 28.6%, P = 0.031)” and have removed non-significant p-values for clarity. (Page 2, line 38-39)

- why conclusion based on the patients’ perspective .. the 1ry outcome as mentioned in the text is the duration of motor recovery assessed objectively.
**Response:** Thank you for your valuable comment. The conclusion is based on the patients' perspective because the recovery of motor power was self-assessed and documented by the patients themselves. This method of measurement reflects their subjective experience and aligns with the study's aim to evaluate outcomes from the patient's point of view. However, we have changed the conclusions for more clarity. (Page 2, line 41-43)

3- in methods:
-anesthesiologist with expertise in regional anesthesia .. add the number of experience years
**Response:** Thank you for your valuable comment. We added “more than five years” in Anesthetic protocol of Materials and Methods section. (Page 7, line 129-130)

- why the motor block was not assessed from the onset of motor block till recovery! including the surgery duration.
**Response:** Thank you for your valuable comment. We apologize for any misunderstanding in our description. In fact, the duration of the motor block was measured by the time the block is completed until the patient feels that motor power has returned to preoperative levels. We have revised and clarified this in the manuscript. (Page 8, line 155-158)

- regarding 2ry outcomes
define the time point of measurement for each parameter and the method used for measurement.
**Response:** Thank you for your valuable comment. The secondary outcomes were 1) the time to the first pain- when the patient feels pain in the surgery area for the first time, 2) worst pain scores, 3) average pain scores, 4) pain medication usage, 5) patient satisfaction scores, 6) desired speed of motor recovery- the amount of time a patient needs to recover their motor block after surgery, and 7) the rebound pain- the pain score increased more than 2 points from the average pain score. (Page 8, line 159-163)

also mention which pain medication dose you follow up?
**Response:** Thank you for your valuable comment. The pain medications assessed during follow-up varied among patients. Details regarding the pain medications were described in of the Anesthetic Protocol section in the Materials and Methods. (Page 8, line 147-152)

mention the frequency you followed up the pain scores during a specified duration.
**Response:** Thank you for your valuable comment. The study was planned to include ambulatory and inpatient cases. In their blocked diary, we asked the patients to note the time to motor recovery, the time to first pain, and the worst and average pain scores in the first 24 hours. A single-blinded assessor, who was a member of the research team, contacted patients on the evening of the surgery and again the following evening to collect data. Pain scores were assessed by asking patients to report their average pain score throughout the day and their worst pain score, without specifying a particular time interval for individual assessments. (Page 9, line 164-172)

what is the difference between patient satisfaction scores, and the patient’s preferred duration for motor recovery ??
**Response:** Thank you for your valuable comment. We want to present the patient's satisfaction score and their desired speed of motor recovery after surgery to emphasize the importance of motor recovery in this type of patient. The "desired speed of motor recovery" refers to the ideal time frame the patient envisions or desires for motor recovery to occur after surgery. In contrast, "patient satisfaction scores" reflect the patient’s overall satisfaction with their experience, which may include factors beyond motor recovery and may not necessarily align with their initially desired recovery duration. (Page 8, line 161-162)

- mention the software used for sample size calculation
**Response:** Thank you. We used the nQuery Advisor 7.0 program for sample size calculation. We’ve already add in the text manuscript. (Page 9, line 179-180)

- mention the level of significance in the statistical analysis.
**Response:** Thank you for your valuable comment. We’ve already added the level of significance in the statistical analysis part of Materials and Methods section as “The statistical significance was defined as a p-value < 0.05”. (Page 10, line 191)

4- in the results:
- the table is inserted after the cited text not before it.
**Response:** Thank you for your valuable comment. We’ve already inserted the table after the cited text.

- according to table 2 :
Axillary block primarily affects the radial, median, and ulnar nerves, with less effect on the musculocutaneous and axillary nerves. So the Tourniquet sites (usually above the elbow) may not be adequately anesthetized. the authors should clarify how they deal with this deficiency in the methods (anaesthesia protocol) **Response:** Thank you for your valuable comment. To address this concern, we used propofol infusion not only to provide sedation but also to alleviate ischemic pain caused by tourniquet pressure. We have revised and clarified this in the text. (Page 8, line 144)

6. PLOS authors have the option to publish the peer review history of their article ([what does this mean?](https://journals.plos.org/plosone/s/editorial-and-peer-review-process#loc-peer-review-history)). If published, this will include your full peer review and any attached files.

If you choose “no”, your identity will remain anonymous but your review may still be made public.

**Do you want your identity to be public for this peer review?** For information about this choice, including consent withdrawal, please see our [Privacy Policy](https://www.plos.org/privacy-policy).

Reviewer #1: No

Reviewer #2: No

Reviewer #3: No
